# Services of Donald Macleod

**Published:** 1843-07-01

**Authors:** 


### OBITUARY.

### SERVICES OF DONALD MACLEOD, M.D., LATE INSPECTOR-GENERAL OF HER MAJESTY'S HOSPITALS IN THE EAST INDIES.

SINCE the peace of 1815, hardly a single death has occurred, of any officer of any merit or service, that has not been followed by some biographical notice in some of the periodical prints of the metropolis.

Death has not spared the KG.'s—the KT.'s—KP.'s—GCB.'s—KCB.'s—GCMG.'s—KCMG's—CMG.'s—GCH.'s—KCH.'s—KH.'s, or last, though not least, the W's, or granted to them any immunity from the common doom, in consideration of their previous services; and, if the customary three vollies of musketry have not sounded their requiem, they have seldom wanted trumpeters, in the shape of memoir-writers to proclaim their praises.

It is far different with the unobtrusive but the important services of the Naval or Military Surgeon. To him the votive urn is seldom erected. No glorious blazonry closes *his* prospect. With him there is no spirit-stirring association of ideas with the camp or the field; no anticipation of medals or honors. He forms in himself a moral anomaly, since to his toils, labours, services and merits, is denied what is so lavishly conceded to those of his brother officers of every military arm. He is the humiliating exception to the rule of—

Palmam qui meruit ferat:—

medals, stars, military insignia, are not for him, nor the fond hope—

" Of being remembered in his line
In his land's language."

Ought this stigma upon an useful and an honorable class to continue in force one hour longer? We leave it to every man of common sense, and possessing a knowledge of the first principles of justice, to answer the question.

The surgeons of our fleets and armies, even the most distinguished, have sunk into obscure graves, like the most ordinary citizens, uneulogised and even unnoticed. Nevertheless would it be most easy to shew, that in the hour of peril, in what Napoleon emphatically called "*two o'clock in the morning courage,*" which he knew but few to possess; in the noble fortitude, in the energy, enterprize, and strength of will, necessary to meet and to conquer danger; these have not been behind the best and foremost of their countrymen, whether at sea or on shore. Amongst the best—amongst the most modest, the kindest and the bravest, of the class we are here speaking of, was Donald Macleod. A native of the Isle of Skye, he entered the Army at a time when its ranks were crowded with his fellow-isles-men,* many of whom have since been variously distinguished in every quarter of the globe.

---

* Within the last 40 years, the Isle of Skye, barren though its soil and scanty

Before we present to the reader the brief but touching memorial presented in 1827 by Dr. Macleod to the Duke of Wellington, we will notice an incident in his life which, at the same time that it shocked the feelings, excited the lively sympathies of the army. We believe it was at the siege of St. Sebastian that the subject of this notice was conversing with a younger brother, a captain on duty in the trenches, when a cannon-shot literally knocked this latter officer to pieces, covering the elder brother with his blood and brains.

We knew the late Dr. Macleod intimately, but never heard him allude in the remotest way to this event. The following we received from him while on his last honorable and important service in India. It was given us with a modest smile, characteristic of the kind-hearted and estimable author, than whom we have not known a better man or a warmer friend, and our career has been long and varied.

*Memorandum of the Services of Staff-Surgeon Donald Macleod, M.D.*

| | |
|---|---|
| Hospital Assistant .. .. .. .. | 10th Sept. 1799. |
| Assistant-Surgeon 82d Regt. .. .. | 30th June, 1800. |
| Surgeon 38th Regiment .. .. .. | 25th Nov. 1803. |
| Staff-Surgeon .. .. .. .. .. .. | 24th Sept. 1813. |
| Placed on Half-pay .. .. .. .. | 25th April, 1821. |
| Re-appointed .. .. .. .. .. .. | 15th March, 1827. |

Previously to entering the regular service, I was Surgeon's Mate of the 1st Battalion and Surgeon of the 2nd Battalion of the Breadalbane Fencibles, from the 25th February, 1798, to 24th March, 1799. I served with the Duke of York in Holland, in 1799. In the Mediterranean to the end of the war in 1802. At the capture of the Cape of Good Hope, under Sir David Baird. At Monte Video, under Sir Samuel Auchmuty; and at Buenos Ayres, under General Whitlock. I went to Portugal with Sir Arthur Wellesley, and was at all the operations of that campaign to the battle and embarkation at Corunna.

I was at Walcheren during the whole time it was occupied, and returned to the Peninsula in May 1811. I was in the battles of Salamanca and Vittoria, and at the sieges of Burgos and St. Sebastian, and in all the actions fought in the neighbourhood of Bayonne and Orthes. I embarked at Bourdeaux for Canada, with the Brigade commanded by Major-General Robinson, and served there for a year; during which period I was present at Platsburg, and at most of the operations on the Lower Frontier. On my return, I joined the army in France, and remained there till the formation of the army of occupation.

I did duty at Dover, and in London, from June 1816 to May 1821. On retiring on half-pay I went to New South Wales with the Governor, Sir Thomas Brisbane, where I remained about four years; and although not absolutely employed in the Medical Department of the Army, I was still in the King's Service.

I am now returning as Staff-Surgeon to that colony, without one single advantage, and with nothing but the bare pay of the rank I have held for twenty-four years, in every quarter of the globe. This is my position after a service of 29 years and 4 months.

DONALD MACLEOD, M.D.

*London*, 15*th Aug*. 1827. *Surgeon to the Forces.*

---

its population, furnished to the public service 21 general officers, 45 colonels, 600 majors, captains, and subalterns, 10,000 foot soldiers, 4 governors of colonies, 1 governor-general, 1 adjutant-general, 1 chief baron of England, 1 judge of the Supreme Court of Scotland, besides many other superior officers.—*Scotch newspaper*. To which it may be added, that to the learning and worth of some of its clergy the celebrated Dr. Samuel Johnson has warmly testified.

There is little to add to the above statement. I sailed from the Cove of Cork on the 27th September, (being the fifth time I left that place for Foreign Service,) and landed at Sidney on the 30th January, 1828. I remained as Staff-Surgeon, and principal medical officer of the Colonies of New South Wales and Van Diemans Land to the 17th January, 1830, when I sailed for Bombay, having been appointed Deputy-Inspector-General, and remained in that Presidency till February 1834, when I was transferred to Madras, and from thence promoted to be Inspector-General in Bengal in July, 1837.

DONALD MACLEOD.

Such is the plain unvarnished statement of one who, had he belonged to any other class but that of which he was so distinguished an ornament, would have had his name enrolled among those whom (to quote the sacred historian)—"the king delighteth to honour." What! perhaps some unreflecting objector will say—did he not attain the highest rank of his corps—did he not die an Inspector-General of Hospitals? Very true—but does the human mind aspire to nothing beyond the mere heritage of routine? Would a general officer, or even a colonel of a regiment, be satisfied were the same argument to be applied to him? If *he* would not, wherein is the justice of supposing that the medical officer either is, or *ought* to be, contented with the mere status that every average man may obtain? Are we never to rise beyond shop-keeper ideas, such as "the foremost man of all the world"—Napoleon, reproached us with? Are we never to get out of the slough of mere money considerations in recompensing merit of a high order? What was it that made the French army prompt and ardent as one man to follow their leader to danger and death? Our reply is simple—the assurance of renown living or dead, and the electric fire of that admirably devised machine for rousing emulation, and keeping the current of human nature ever clear and flowing—the legion of honor! It is not enough then that for such a person as the subject of this memoir his country should have done no more than make him an Inspector-General of Hospitals. Up-hill as the task may be, we do not yet despair to see the claims of a too-long neglected class placed upon a broader and more honorable basis than they have ever been in this country, which has been contented in this as in some other things having scientific reform in view, to halt behind France.

We have said that the subject of this sketch was a kind and brave man. This however is only giving a very meagre outline of his character, and his rare modesty rendered it difficult for the observer to procure elements for filling in the portrait, save when they evolved themselves unconsciously in the slipshod easy intercourse of private life, with friends who loved and valued him. As a professional man he was quietly but keenly and accurately observant, and took a deep interest in all that related to the healing art. His large experience, and his entire freedom from dogmatism, with the admirable good sense which was ever in him a preservative against the allurements of theory, made his conversation as instructive as it was pleasant. His reading was not confined to the professional walk, but comprehended an intimacy with the best historical authors, and the works of the most distinguished writers in dialectics and general science. It required however, that those with whom he conversed should, as the phrase is, draw him out, when his extensive acquaintance with many men and countries afforded him a facility of illustration, and a diversity of *fresh* anecdote, which he whose travels are limited to a library, or fire-side scenes can never command. His knowledge of *the soldier*, individually and collectively, of his capacity, of his weak and of his strong points, ill or in health, was intuitive and profound. To the last he was no less respected than beloved by the men and their wives—for though strict and firm in the exercise of professional authority, he tempered it with a kindness and consideration that endeared him to them all, as a friend conscientiously interested in their welfare. It may enter very little into the calculations of a general or commandant, but it is nevertheless most true, that in the statistics of armies, the bearing, the conduct, and the tenderness no less than the promptitude, skill, and scientific resources of military surgeons, have an immense effect in cherishing good feeling, and preserving and strengthening discipline among the soldiery. This will become more obvious when it is recollected that there are only two officers in a regiment who may be supposed to have a general knowledge of the men in a state of health, viz. the commandant and the adjutant. The acquaintance of the remaining officers is confined to the men of their own companies. The military surgeon, on the other hand, comes in contact with every man in the regiment by turns, for at some time or other they all may be said to pass through his hands. His knowledge of their character therefore must be more esoteric, so to speak, than that of their officers, seeing that he has, as it were, the measure of their moral power to endure suffering and privation: for suffering and privation are the conditions upon which the soldier comes under the military surgeon's care. The family relations of the soldier also bring him peculiarly within the sympathy of the military surgeon. When writhing under the lash, or pining on the cot where he lies wounded or dying, it is to the military surgeon that he looks for succour and consolation. The military surgeon who understands his duty, and acts up to its solemn obligations, is not only looked up to as an officer that must be obeyed, but as an advocate, a conciliator, and a friend in a variety of ways, that any one who has served even for a short time with troops, will at once comprehend, without rendering it necessary for us to dilate further upon it here.

By his brother officers, of every arm, Dr. Macleod was universally beloved and esteemed for the qualities of his head and heart. In his personal appearance, he was about five feet ten inches high, and of compact mould. His countenance was of the contemplative cast, and peculiarly expressive, more especially when lightened up by a smile. His capacity for enduring fatigue and vicissitudes of temperature, was extraordinary. With him it was no uncommon thing to pass night after night without sleep, refreshment, or changing his clothes, even when the weather was wet and inclement. Of active habits, and temperate no less in diet than in recreation, what many might deem hardships, were regarded by him as trifles. In this he evinced himself a genuine highlander of the old stamp, in whom self-control and powers of uncomplaining and cheerful endurance, were united with gentlemanly courtesy, and knowledge of the world. Perhaps it had been well had his constitutional and soldierly indifference to fatigue and changes of climate, been less. In that case, perhaps, a greater solicitude for self, than formed with him a trait which abounds in our day, would have led to the prolongation of his valuable life. Always ready at the call of duty, to move wherever his services might be required, he had no time to consolidate those securities for ease and comfort, that may be commanded by officers of a less shifting and more influential department. It was, therefore, nothing for him to be on the route, one day, for the snowy Himalayahs, and anon for the burning plains of Bengal. This most probably affected his health more than he would willingly allow, until fairly knocked down by positive illness. Ever more solicitous about the health of others than his own, the insidious approach of the last enemy were not sufficiently foreseen or guarded against. It might be said of him, that his complaint consisted less of positive ailment, than of a negation of his usual habits. First came an indefinite languor, attributed to temporary causes, and hoped to be got over "in a day or two." Then came loss of appetite, and a scarcely perceptible wasting or loss of flesh. Next became observable a certain quickening of pulse, a febrility, so to speak, and irregularity of hepatic and gastric functions. Such were the wily approaches of liver disease—and ere he appeared himself aware of the danger, the enemy was in the citadel. With the hope of benefiting by change of air, he proceeded in a boat upon the Calcutta River—and had the advantage of the best medical aid—but all, alas! was without avail. He was latterly perfectly aware of his own state—and the constant presence in the expectoration of purulent and bloody sputa from an abscess that burst into the cavity of the thorax—to less experienced and shrewd observation than his own, would have told the tale with sufficient plainness.

He was in the possession of his faculties to the last moment, and even when the exhaustion of the moribund state made him too weak to speak, he still evinced by signs that he was perfectly calm, collected, and resigned. Thus, a little before he expired, on a spoonful of restorative mixture being offered to him, he waved his hand, with a mournful smile, that spoke as well as words, "It is of no use."

